# Acute Exposure to Bisphenol A Causes Oxidative Stress Induction with Mitochondrial Origin in *Saccharomyces cerevisiae* Cells

**DOI:** 10.3390/jof7070543

**Published:** 2021-07-07

**Authors:** Ivana Ďurovcová, Eduard Goffa, Zuzana Šestáková, Dominika Mániková, Katarína Gaplovská-Kyselá, Miroslav Chovanec, Andrea Ševčovičová

**Affiliations:** 1 Department of Genetics, Faculty of Natural Sciences, Comenius University, Ilkovičova 6, Mlynská Dolina, 842 15 Bratislava, Slovakia; durovcova4@uniba.sk (I.Ď.); katarina.gaplovska@uniba.sk (K.G.-K.); 2 Biomedical Research Center, Department of Genetics, Cancer Research Institute, Slovak Academy of Sciences, Dúbravská cesta 9, 845 05 Bratislava, Slovakia; eduard.goffa@savba.sk (E.G.); zuzana.sestakova@savba.sk (Z.Š.); dominika.manikova@savba.sk (D.M.); miroslav.chovanec@savba.sk (M.C.)

**Keywords:** bisphenol A, nuclear genome integrity maintenance, ROS production, DNA oxidative damage, protein carbonyl content, mitochondrial involvement

## Abstract

Bisphenol A (BPA) is a major component of the most commonly used plastic products, such as disposable plastics, Tetra Paks, cans, sport protective equipment, or medical devices. Due to the accumulation of excessive amounts of plastic waste and the subsequent release of BPA into the environment, BPA is classified as a pollutant that is undesirable in the environment. To date, the most interesting finding is the ability of BPA to act as an endocrine disrupting compound due to its binding to estrogen receptors (ERs), and adverse physiological effects on living organisms may result from this action. Since evidence of the potential pro-oxidizing effects of BPA has accumulated over the last years, herein, we focus on the detection of oxidative stress and its origin following BPA exposure using pulsed-field gel electrophoresis, flow cytometry, fluorescent microscopy, and Western blot analysis. *Saccharomyces cerevisiae* cells served as a model system, as these cells lack ERs allowing us to dissect the ER-dependent and -independent effects of BPA. Our data show that high concentrations of BPA affect cell survival and cause increased intracellular oxidation in yeast, which is primarily generated in the mitochondrion. However, an acute BPA exposure does not lead to significant oxidative damage to DNA or proteins.

## 1. Introduction

Phenolic compounds are chemically defined as compounds containing hydroxylated aromatic rings, which comprise the hydroxy group attached directly to the phenyl, substituted phenyl, or other aryl groups. Bisphenol A (BPA) is a white crystal substance produced commercially by strong acid-catalyzed condensation of phenol and acetone under mild conditions of temperature and pressure [1]. Nowadays, BPA is a chemical compound abundantly used in the manufacture of many plastics, such as some plastic bottles, medical disposable devices, sport protective equipment, surface coatings, or daily used disposable products, for example, straws, cutlery, plates, or cups [2,3,4,5]. The fact that plastic products are currently overproduced, very persistent, durable, and that BPA can leach from the material used, leads to a state that BPA residues accumulate in all components of the environment, especially in soil and water systems [6,7]. From waste landfills or during the disposal processes, trace amounts of BPA can migrate directly into water sources [8]. Additionally, BPA can be transmitted from food containers, plastic bottles, or dental fillings into the digestive system [3,9]. BPA can also serve as a heat-activated developer when added as a powder to thermal paper [10,11], and then, 8.6% of the BPA attached to the paper can penetrate through the skin into the body, as has been shown both in vitro and in vivo. This dermal exposure represents a minimal contribution to the total human body’s exposure to BPA [12,13].

Migration of BPA from polycarbonate plastics can be influenced by different factors, including length of food storage time (direct contact of food with coating material), composition of food, temperature (the higher temperature, the higher BPA release) [14,15], ultraviolet light, and the age of the plastic itself [16]. The release is based on the hydrolyzation of the ester bonds by these factors, where free molecules of BPA are transferred to the food or environment [17].

Over time, BPA has become ubiquitous, and more scientific teams around the world have become interested in its effects on living organisms. To date, the most important findings show that BPA has the ability to act as an endocrine disruptor [18]. In higher organisms with a developed endocrine system, hormones link the nervous system and bodily functions, such as development, growth, metabolism, immunity, reproduction, and behavior. Overall, endocrine disruption is a process, in which chemicals from the environment or of internal body origin may interfere with the hormonal system, and thereby may be harmful to the organism. With a structure similar to estrogen, BPA can relatively easily bind to estrogen receptors (ERs), precisely its two subtypes (ERα and ERβ) [19,20], or to a transmembrane G protein-coupled receptor 30 (GPR30) [21]. Consequently, BPA is able to interfere with a lot of fundamental functions in an organism, which may lead to several diseases or developmental defects [18], for example, heart disease or heart valve leaflet defects [22], neurodegenerative diseases (via reduction of neural stem cell proliferation and differentiation in the hippocampus and subventricular zone of the brain) [23], defects in adipogenesis, metabolic disorders [24,25], or sperm quality and quantity impairment [26].

The minor mechanism of BPA action on living cells appears to be caused by oxidative stress. Excessive production of reactive oxygen species (ROS), in some cases accompanied by depletion of the cell’s antioxidant defense system (including decreased activity of the antioxidant enzymes, such as superoxide dismutase, catalase, or peroxidase), may eventually lead to the induction of DNA or protein damage, or inhibition of cell division [27,28,29]. Protein carbonyl derivatives are formed by an oxidative attack on some amino-acid side chains [28]. As compared with other oxidative modifications, carbonyls are relatively difficult to induce and carbonylation is an irreversible oxidative process [30]. In addition, BPA can significantly reduce the enzyme activity of mitochondrial electron transport chain complexes along with a significant increase in lipid peroxidation and protein oxidation status [31]. Moreover, a significant mitochondrial dysfunction including ROS overproduction, mitochondrial membrane hyperpolarization, lipid peroxidation, and the release of proinflammatory cytokines after BPA treatment have all been demonstrated [32]. In addition, BPA displays negative effects in many living systems [22,23,25,33,34,35,36]. Increased ROS levels were detected in several tissues of bighead carp fish (*Aristichthys nobilis*) [33], and also in murine macrophages [34]. According to Adamakis et al. [35], BPA exerted acute anti-mitotic effects on meristematic root-tip cells of *Pisum sativum*, and microtubules represented the primary target of the BPA toxicity. Interestingly, BPA application can result in a significant increase of non-regulated energy loss in the photosystem II in the leaves of the seagrass *Cymodocea nodosa* [36].

Human biomonitoring and epidemiology studies have focused on the long-term effects of the environmental pollutant BPA on a wide range of cells and organisms, from the simplest through different classes of vertebrates to the most complex―human cells derived from different tissues. In the present study, we focused on the acute effects of environmentally relevant and high doses of BPA on *Saccharomyces cerevisiae* cells, a commonly used genetic model system that provides a fast, inexpensive, and undemanding possibility of cultivation, and thus quickly obtained answers to scientific questions. This model system is particularly suitable for studies underlying the effects of BPA because it lacks ERs, thereby, allowing dissecting endocrine disruptor-dependent and -independent effects of this compound. The main objective of this study was to investigate the ability of BPA to induce ROS in yeast cells, to localize the generated ROS within the cell, and to reveal ROS targets.

## 2. Materials and Methods

### 2.1. Cell Cultivation

*Saccharomyces cerevisiae* WT strain YW465 (*MATα*, *ade2Δ0*, *his3Δ200*, *leu2-LYS2*, *met15Δ0*, *trp1Δ63*, and *ura3Δ0*) was used. In addition, another WT strain (ρ^+^) RDKY3615 (*MATa ura3-5*, *trp1Δ63 his3Δ200 leu2Δ1 lys2ΔBgl hom3-10 ade2Δ1 ade8 hxt13::URA3*) and its isogenic derivative lacking mtDNA (ρ^0^) ZDY3 (RDKY3615 *mgm101::LEU2*) [37] were used. The cells were grown in liquid YPD medium (1% *w*/*v* yeast extract, 2% *w*/*v* peptone, 2% *w*/*v* glucose, and 2% *w*/*v* agar for plates) overnight (O/N) at 29 °C with appropriate shaking. An O/N culture was used to inoculate fresh medium, and the cells were grown until an exponential growth phase was reached. Afterward, the cells were washed with, and resuspended in, 50 mM phosphate buffer (1 M KH_2_PO_4_, 1 M K_2_HPO_4_, pH 7.8) to a concentration of 4 × 10^8^ cells/mL for PFGE, or 2 × 10^8^ cells/mL for other analyses.

### 2.2. BPA Treatment

BPA treatment (in all experiments except the cell growth) was performed at room temperature (RT) in a phosphate buffer (non-growing conditions) with agitation for 3 h. The treatment consisted of environmentally relevant (1, 10, and 100 µM) and high concentrations (500 and 1000 µM) of BPA. Since 1% dimethyl sulfoxide (DMSO) was used to dissolve BPA, it was also added to untreated cells to achieve the same DMSO content in all samples tested. Because neither phosphate buffer nor 1% DMSO affected cell viability, 1% DMSO was used as a relevant control in all analyses.

### 2.3. Cell Growth

The cells in the exponential growth phase were inoculated into fresh liquid YPD medium supplemented with indicated concentrations of BPA to achieve the initial concentration of 5 × 10^5^ cells/mL. Then, aliquots were taken every 2 h and the cell concentration was calculated using a Bürker chamber. In total, cell growth was monitored over a period of 20 h. The growth curve represents the average values of cell concentration from 3 independent experiments.

### 2.4. Cell Survival

The treated cells were washed with, and serially 10-times diluted in, phosphate buffer to a concentration of 1 × 10^4^ cells/mL. Then, 1 × 10^3^ cells from each sample were plated on YPD agar plates. The number of colonies on each Petri dish was counted after 72 h incubation at 29 °C. On the basis of the number of colonies, the cell survival was calculated and normalized to the untreated cells.

### 2.5. Pulsed-Field Gel Electrophoresis

PFGE was performed, as previously described [38]. Briefly, BPA-treated and untreated cells were washed twice in 50 mM EDTA (pH 8.0) at a density of 4 × 10^8^ cells/mL, and the resulting pellets were resuspended in a buffer consisting of 1 M Tris, 1 M NaCl, and 0.5 M EDTA (pH 7.5). The cell suspension was equilibrated to 50 °C and 10 μL of lyticase (10 mg/mL in 0.9 M sorbitol, 0.1 M EDTA, 0.1 M Tris, pH 8.0, Sigma-Aldrich, Kawasaki, Japan) was added. The cell suspension was immediately mixed with an equal volume of 2% agarose for PFGE sample preparation (Sigma-Aldrich) and transferred into plug molds which were kept on ice until the plugs were completely solidified. The cell wall was degraded by incubating the plugs for 1 h at 37 °C in 1 M Tris, 0.5 M EDTA (pH 7.5), supplemented with 85 µg/mL lyticase. Then, the plugs were washed with a buffer of 1 M Tris, 0.5 M EDTA (pH 8.0), and subsequently incubated O/N in 0.5 M EDTA (pH 8.0), 0.2% *w*/*v* sodium deoxycholate, 1% *w*/*v* sodium lauryl sarcosine, and 1 mg/mL proteinase K, at 50 °C. The next day, the plugs were washed twice with a wash buffer consisting of 1 M Tris, 0.5 M EDTA (pH 8.0), with gentle rolling of the tubes. Afterwards, the plugs were incubated with 1 mM protease inhibitor (Pefabloc AEBSF, Roche, Mannheim, Germany) for 2 h at 37 °C and washed again with the wash buffer, with gentle rolling of the tubes. Then, the plugs were washed with 10-times diluted wash buffer and with reaction enzyme buffer for the Fpg enzyme (40 mM Hepes, 0.1 M KCl, 0.5 mM EDTA, 0.2 mg/mL BSA, pH 8.0), with gentle rolling of the tubes. In the next step, the Fpg enzyme was added to one series of samples at a final concentration of 25 U per ½ plug O/N at 37 °C. The second series of samples was stored in the wash buffer at 4 °C until used. The next day, the enzyme reaction was stopped by heat inactivation at 60 °C for 10 min and the plugs were washed with the wash buffer. Electrophoresis was performed in TAE buffer (40 mM Tris-acetate and 1 mM EDTA, pH 8.0) using a CHEF-DR^®^ III Variable Angle System (Bio-Rad, Hercules, CA, USA) with constant voltage 4.5 V/cm for 23 h at 14 °C, angle 120° and a switch time of 60–120 s. After electrophoresis, DNA in the gel was stained with 0.5 μg/mL ethidium bromide for 30 min at RT and subsequently de-stained in water containing RNase A (4 μg/mL) for 1.5 h. After washing the gel with water, an image was taken using the Li-Cor Odyssey^®^ Fc Imaging System 5.x. 

### 2.6. Flow Cytometry Analysis

The treated and untreated cells were washed with, and resuspended in, phosphate buffer, then 10-times diluted and added to 10 μM DCFDA (Sigma-Aldrich) in 1% DMSO. DCFDA, as a lipophilic compound, permeates the metabolically active cell [39]. Once oxidized by ROS, DCFDA is converted to a highly fluorescent DCF, which fluoresces at 527 nm, and thus provides an estimate of ROS levels [40]. Staining was performed at 30 °C in the dark for 1 h without shaking, as previously published [41]. Afterwards, the fluorescent dye was washed off and the cells were resuspended in ice-cold phosphate buffer. To distinguish between the living and dead cells, propidium iodide (PI, Sigma-Aldrich) (1 μL from stock 1 mg/mL) was added to each sample (500 μL) before scoring 5 × 10^4^ cells on a BD FACS Canto II flow cytometer (BD Biosciences, Franklin Lakes, NJ, USA). FITC (530/30 nm) and PE (585/40) filters were used for DCFDA and PI fluorescence, respectively. Since we used fluorescence compensation, we were able to exclude dead cells (PI^+^), and hence KALUZA software (Beckman Coulter, GmbH, Life Sciences, Krefeld, Germany) was used to score the DCFDA fluorescence and to determine the percentage of DCF positive cells in the samples. The measured values were normalized to the untreated cells.

### 2.7. Fluorescent Microscopy

The treated and untreated cells were washed with, and resuspended in, phosphate buffer. To visualize the total ROS levels, cells were added to 10 μM DCFDA (Sigma-Aldrich) in 0.1% DMSO and stained for 60 min at 30 °C in the dark without shaking. To visualize mitochondrial O_2_^•−^, cells were incubated with 5 μM MitoSOX Red (Invitrogen, Thermo Fisher Scientific, Waltham, MA, USA) in 0.1% DMSO for 10 min at 37 °C in the dark without shaking. Afterwards, the fluorescent dyes were washed off and the cells were resuspended in phosphate buffer. Just before imaging, the cells were fixed with 0.1% (*w*/*v*) Poly-L-lysine solution (Sigma-Aldrich). The cells were imaged with an IX83 inverted microscope (Olympus, Tokyo, Japan) equipped with a Planapochromate 60×/1.42 oil objective and CAM-XM10 cooled CCD digital camera. For visualization of ROS using DCFDA, U-FGFP filter cube (with excitation spectrum 460–480 nm and emission spectrum 495–540 nm) was used. For visualization of ROS using MitoSOX Red probe, U-FYFP filter cube (with excitation maximum 510 nm and emission maximum 580 nm) was used. For control of the cell morphology, the cells were imaged in a bright field. For capturing the images, the software CellSens Dimension version 1.11 (Olympus) was used. One hundred cells per sample were observed in each experiment. The relative fluorescence intensity was measured using ImageJ software and determined as the average of the ratio of raw integrated density and area for 100 cells per experiment normalized to the untreated cells.

### 2.8. Mitochondrial Membrane Potential

The treated and untreated cells were washed with, and resuspended in, phosphate buffer. The cell pellets were resuspended in 50 nM MitoTracker Red (Thermo Fisher Scientific, Waltham, MA, USA) staining solution in 0.005% DMSO preheated to 29 °C. The cells were incubated for 30 min at 29 °C. After staining, the cells were washed with, and resuspended in, phosphate buffer. Just before imaging, the cells were fixed with 0.1% (*w*/*v*) poly-L-lysine solution (Sigma-Aldrich). The cells were imaged with an IX83 inverted microscope (Olympus, Tokyo, Japan) equipped with a Planapochromate 60×/1.42 oil objective and CAM-XM10 cooled CCD digital camera. For visualization of mitochondria using MitoTracker Red, a U-FMCHE filter cube (with excitation spectrum 565–585 nm and emission spectrum 600–690 nm) was used. For control of the cell morphology, the cells were imaged in a bright field. For capturing the images, the CellSens Dimension software (Olympus) was used. One hundred cells per sample were observed in each experiment. The relative fluorescence intensity was measured using ImageJ software and determined as the average of the ratio of raw integrated density and area for 100 cells per experiment normalized to the untreated cells.

### 2.9. Protein Carbonylation

The BPA-treated and -untreated cells in the exponential growth phase were washed with, and resuspended in, phosphate buffer. In control experiments performed in parallel and aimed at proving that the detection system is valid to yeast proteins, sodium selenite (SeL) treatment at a concentration range of 10–1000 µM was used. According to the manufacturer’s instructions, cell lysates were prepared using the Y-PER^TM^ Yeast Protein Extraction Reagent (Thermo Fisher Scientific). The protein carbonyl content was detected using the Protein Carbonyl Assay Kit for Western Blot (Abcam, Cambridge, United Kingdom). Then, 10 µL of each reaction (corresponds to 5 µg of proteins) was electrophoretically resolved on 10% SDS-PAGE gel. The resolved proteins were further transferred on an Amersham Protran 0.1 µm nitrocellulose membrane (GE HealthCare, Chicago, IL, USA). After electrotransfer, the membrane was subsequently stained with Revert total protein stain (LI-COR) to assess the total protein content of each sample as a loading control. The total protein signal was detected by the Odyssey Fc Imaging System 5.x (LI-COR). After blocking and incubation with primary anti-DNP and HRP-conjugated secondary antibody, the membrane was incubated with SuperSignal^TM^ West Femto Maximum Sensitivity Substrate (Thermo Fisher Scientific). A chemiluminescent signal was detected by the Odyssey Fc Imaging System 5.x (LI-COR). Signal quantification was done using the Image Studio Lite software version 5.2 (LI-COR), where the intensity of protein carbonylation obtained by chemiluminescence was normalized to the entire 700 nm intensity of the corresponding sample representing the total protein content.

### 2.10. Statistical Analysis

The results are presented as the mean ± standard deviation (mean ± SD) from at least three independent experiments. Student’s t-test was used to analyze the significance of differences between the two sets of data (treated samples vs. non-treated samples) in measuring the growth rate and cell survival, fluorescence signal, flow cytometry, and protein carbonylation data analyses. Significant differences were considered * when *p* < 0.05, ** when *p* < 0.01, and *** when *p* < 0.001.

## 3. Results

### 3.1. BPA Exposure Leads to a Decrease in Growth Rate and Cell Survival in Yeast

**Figure 1:**
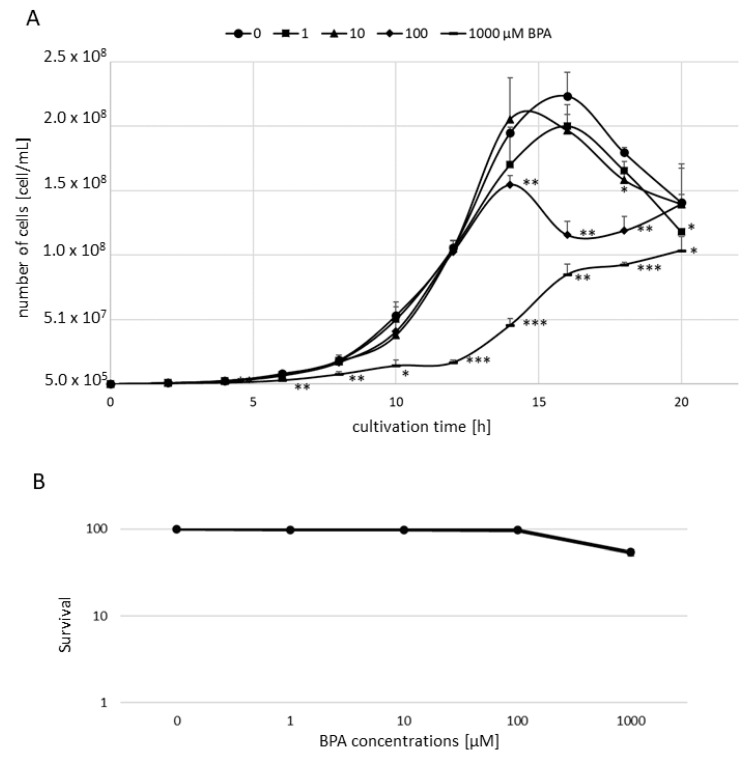
(**A**) Growth rate of *S. cerevisiae* cells after a long time of bisphenol A (BPA) treatment; (**B**) survival of *S. cerevisiae* cells after a short time of BPA treatment. The results are the average of 3 independent experiments. The significance of differences between the means was evaluated by the Student’s *t*-test: * *p* < 0.05; ** *p* < 0.01; *** *p* < 0.001.

To assess the toxic effects of BPA, we monitored the growth rate of yeast cells after long, and the viability after short exposure to this compound. As evident in Figure 1A, such treatment conditions lead to a statistically significant decrease in the growth rate at 1000 µM BPA concentration. At 100 µM concentration, BPA significantly slowed cell growth from a cultivation time of 14 h. Lower BPA concentrations had virtually no slowing down effects on the growth rate. In terms of cell survival, 3 h at 500 and 1000 µM BPA exposure decreased cell viability to 84.1% (*p* < 0.05) and 53.9% (*p* < 0.001), respectively (Figure 1B).

### 3.2. Acute BPA Exposure Does Not Cause DNA Double-Strand Break Induction in the Yeast Nuclear Genome

**Figure 2:**
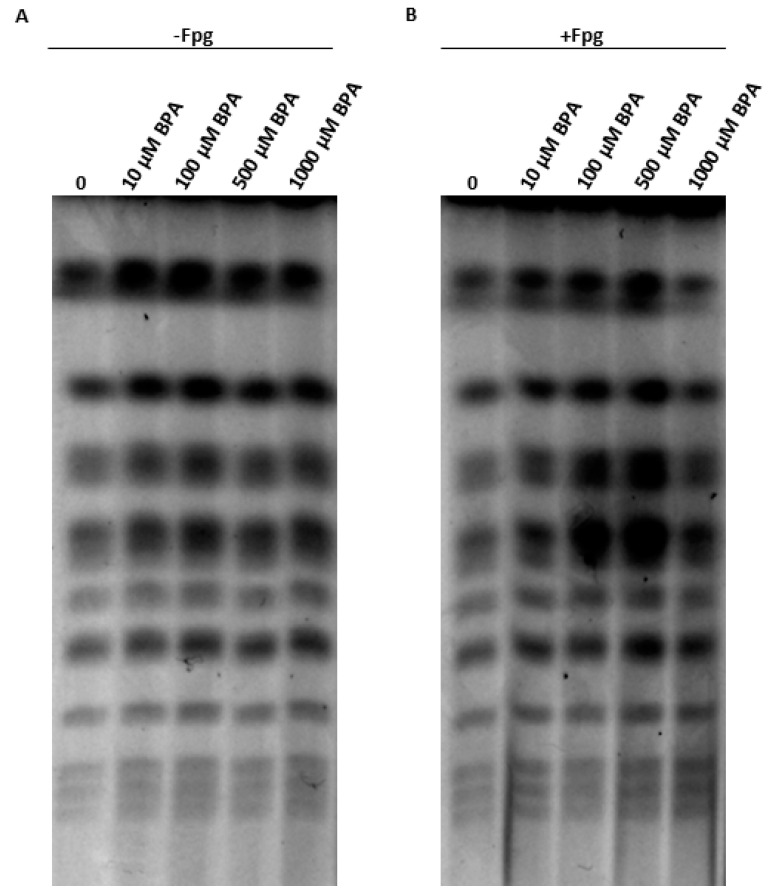
The effect of acute BPA treatment on nuclear genome integrity of yeast cells, as measured by pulsed-field gel electrophoresis (PFGE). The cells were first exposed to increasing concentrations of BPA, and then treated with no (**A**) or 25 U Fpg (**B**) enzyme. Representative gels are shown.

Pulsed-field gel electrophoresis (PFGE) uses a change in the direction of the electric current to separate high molecular weight DNA molecules, such as individual chromosomes. If DNA strand breaks occur, the DNA molecules become fragmented, and smears instead of bands are observed in the agarose gels [42]. Using this method, next, we examined DNA double-strand break (DSB) induction in yeast nuclear DNA after acute BPA exposure. We found that BPA, in the concentration range tested, formed virtually no DSBs in *S. cerevisiae* cells (Figure 2A). After treatment of the agarose-embedded cells with the *Escherichia coli* Fpg enzyme recognizing the oxidized purines and introducing single-strand breaks (SSBs) into DNA, we observed a subtle increase in DNA double-strand breakage, suggesting that BPA induces base damage, to some extent, in yeast nuclear DNA (Figure 2B).

### 3.3. Acute Exposure to BPA Leads to a Rapid Increase of ROS Levels in the Yeast Cells

**Figure 3:**
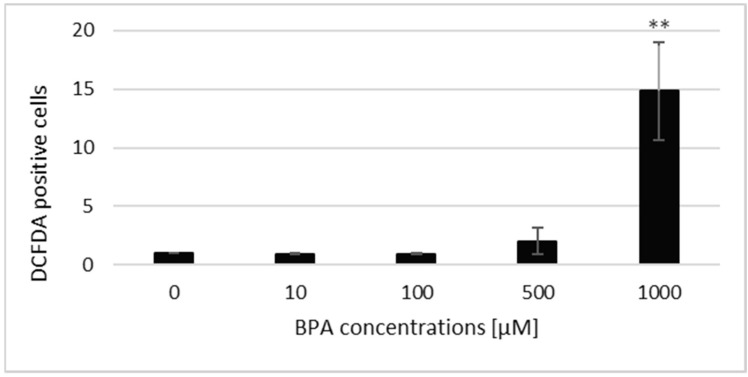
Reactive oxygen species (ROS) induction after BPA treatment in *S. cerevisiae* cells evaluated by flow cytometry. The results are the average of 4 independent experiments. The significance of differences between the means was evaluated by the Student’s *t*-test: ** *p* < 0.01.

The flow cytometry analysis was used to examine the possibility of ROS induction by acute BPA exposure. As shown in Figure 3, BPA at 1000 µM concentration dramatically increases the amount of ROS in yeast cells, i.e., the total ROS levels increased almost 15 times (*p* < 0.01). At the other concentrations tested, we did not notice any significant changes in the ROS levels as compared with the untreated control.

**Figure 4:**
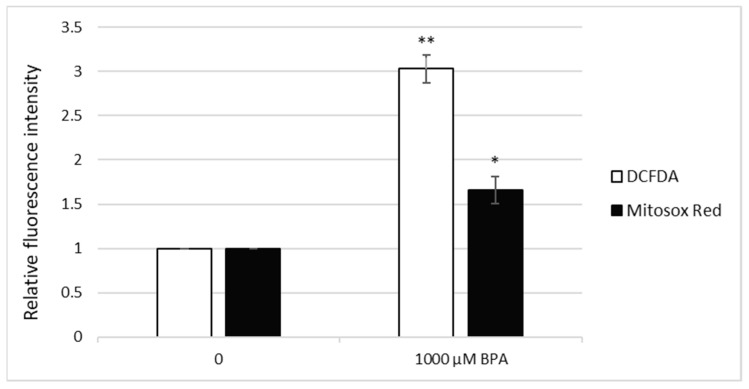
ROS production in *S. cerevisiae* cells evaluated by fluorescence microscopy. While 2′,7′-dichlorofluorescein diacetate (DCFDA) was used to visualize the total ROS levels in a cell, MitoSOX Red selectively detected mitochondrial superoxide anion (O_2_^•−^). The relative fluorescence intensity was measured as the mean value of 100 cells normalized to untreated control. The results are the average of 3 independent experiments. The significance of differences between the means was evaluated by the Student’s *t*-test: * *p* < 0.05; ** *p* < 0.01.

**Figure 5:**
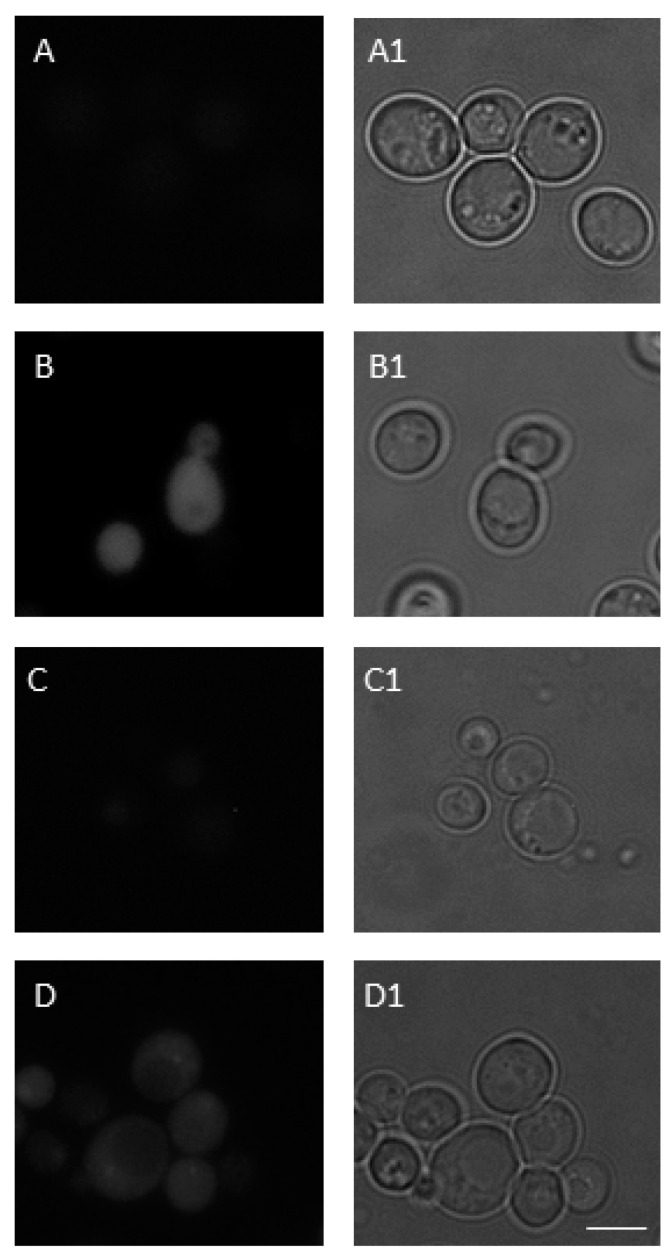
The total ROS and mitochondrial O_2_^•−^ levels after 1000 µM BPA treatment in yeast. Untreated (**A**,**C**) and 1000 µM BPA treated (**B**,**D**) *S. cerevisiae* cells were stained with DCFDA and MitoSOX Red to visualize the total ROS in a cell (**A**,**B**) and O_2_^•−^ generated in mitochondria (**C**,**D**), respectively. Fluorescence (**A**–**D**) and bright field (**A1**–**D1**) microscopy were used for imaging of the cells. Scale bar in micrographs = 5 μm. Representative images are shown.

Thereafter, fluorescence microscopy was used to further extend flow cytometry studies. In line with flow cytometry data, 1000 µM BPA induced increased levels of ROS in *S. cerevisiae* cells (Figure 4 and Figure 5A,B). The use of the MitoSOX Red probe allowed us to specify the ROS that caused this effect. It was clearly shown (Figure 4 and Figure 5C,D) that superoxide anion (O_2_^•−^) produced by mitochondria was the ROS primarily induced by acute BPA exposure.

### 3.4. ROS Level Does Not Increase in Cells Lacking mtDNA

**Figure 6:**
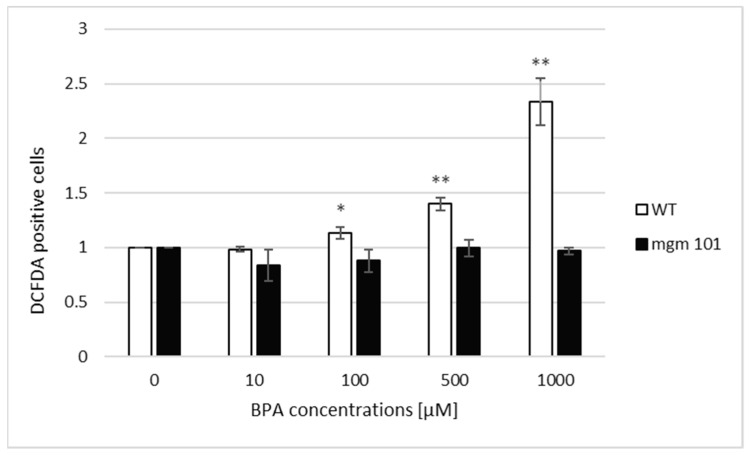
The total ROS level in ρ^+^ (WT) and ρ^0^ (*mgm 101* mutant) cells after BPA treatment evaluated by flow cytometry. The results are the average of 4 independent experiments. The significance of differences between the means was evaluated by the Student’s *t*-test: * *p* < 0.05; ** *p* < 0.01.

To confirm the role of mitochondria in ROS production after BPA treatment, we measured the total ROS levels in cells with (ρ^+^) and lacking (ρ^0^) mitochondrial DNA (mtDNA) by flow cytometry. As expected, the total ROS levels increased 1.13-fold (*p* < 0.05), 1.4-fold (*p* < 0.01), and 2.34-fold (*p* < 0.01) after 100, 500, and 1000 µM BPA treatment, respectively, only in the cells containing mtDNA. In contrast, ROS levels remained unchanged in the cells lacking mtDNA (Figure 6).

### 3.5. Acute Exposure to BPA Leads to a Decrease in Mitochondrial Membrane Potential

**Figure 7:**
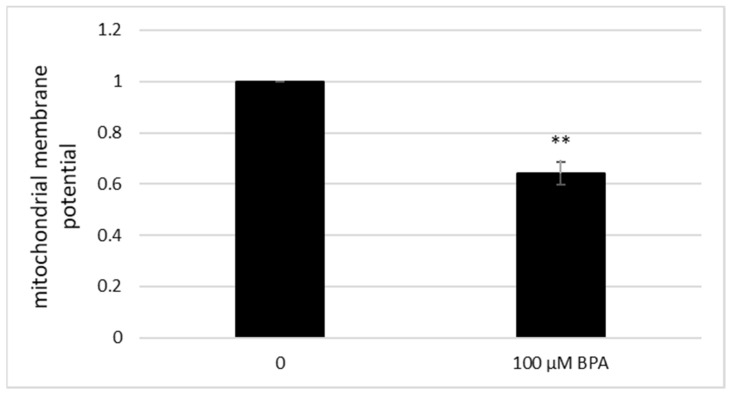
The reduction of mitochondrial membrane potential (MMP) after the 100 μM BPA treatment in *S. cerevisiae* cells. The relative fluorescence intensity was measured as the mean value of 100 cells normalized to an untreated control. The results are the average of 3 independent experiments. The significance of differences between the means was evaluated by the Student’s *t*-test: ** *p* < 0.01.

**Figure 8:**
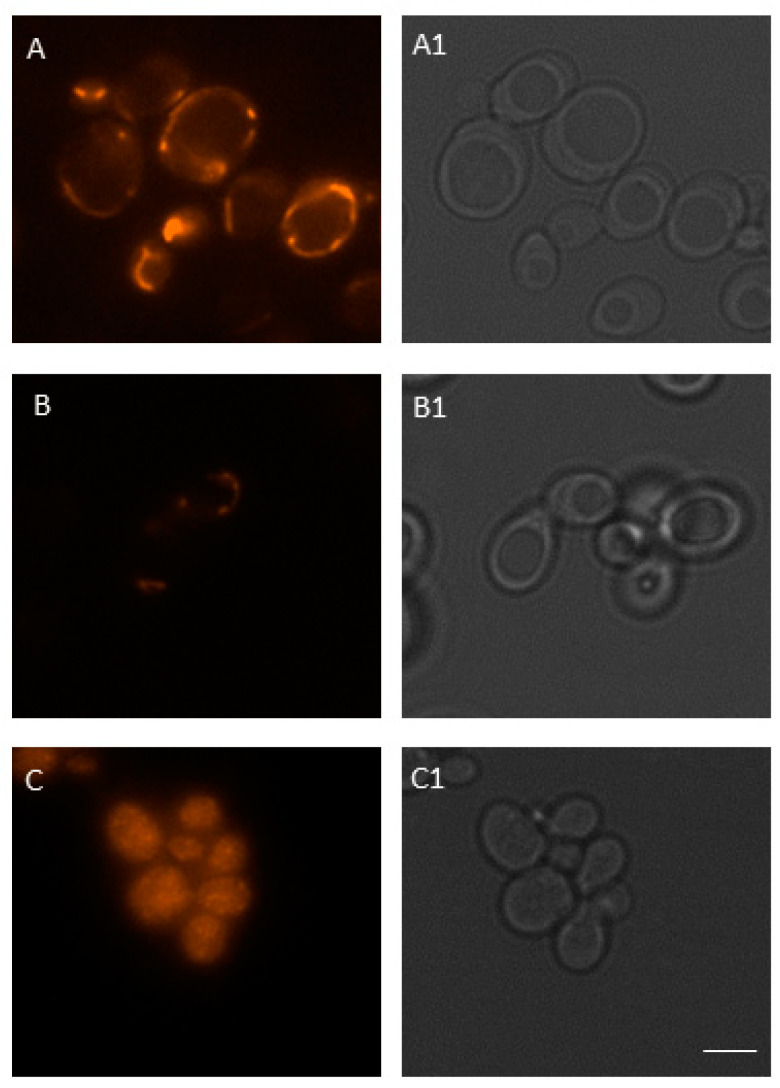
The fluorescence analysis of MMP after BPA treatment in *S. cerevisiae* cells. Untreated (**A**) and 100 (**B**) or 1000 µM (**C**) BPA-treated cells were stained with MitoTracker Red probe to visualize mitochondria. Fluorescence (**A**–**C**) and bright field (**A1**–**C1**) microscopy were used for imaging of the cells. Scale bar in micrographs = 5 μm. Representative images are shown.

To evaluate the mitochondrial status of BPA-treated cells, we visualized the cells using fluorescence microscopy. The 100 μM BPA treatment reduced the mitochondrial membrane potential (MMP) of *S. cerevisiae* cells (Figure 7 and Figure 8B) to 64.15% (*p* < 0.01) as compared with untreated cells. The BPA concentration of 1000 µM had the cytotoxic effect on the cells, as we only detected cells with significantly altered mitochondrial morphology. These cells were most likely dead (Figure 8C).

### 3.6. Acute BPA Exposure Does Not Change the Protein Carbonyl Content

**Figure 9:**
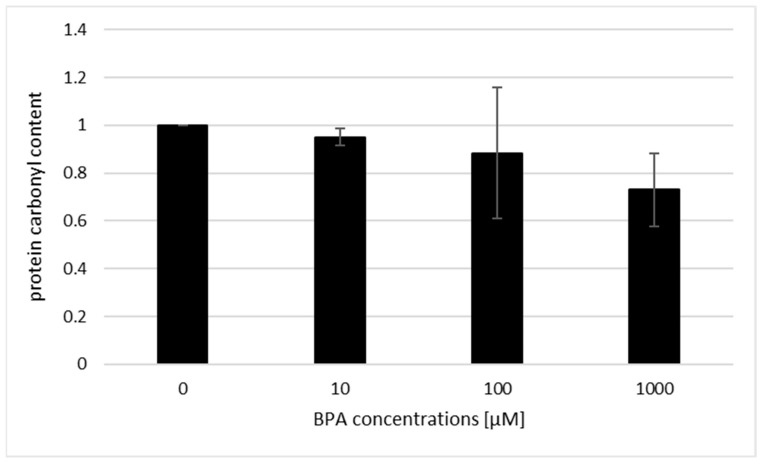
The protein carbonylation after acute BPA treatment in *S. cerevisiae* cells. The results are the average of 3 independent experiments.

Next, we examined the effect of BPA on the protein carbonyl content using Western blotting and cell lysates derivatized with 2,4-dinitrophenylhydrazine (DNPH). It was obvious (Figure 9) that acute BPA exposure virtually did not affect the level of protein carbonylation. To justify this conclusion, a set of control experiments was performed in parallel. Most importantly, the conditions highly expected to increase protein carbonylation, i.e., sodium selenite (SeL) treatment, were used to prove that the detection system used herein was valid to yeast proteins. SeL is a compound that acts as a strong oxidizing agent in *S. cerevisiae* cells and causes increased levels of ROS [38,43]. According to the SeL data (Figures S1–S3), we can undoubtedly see that the carbonylation detection system works for yeast proteins, and therefore we can conclude that BPA has no effect on the protein carbonylation level.

## 4. Conclusions

We demonstrate that BPA at high(er) doses and acute exposure induces oxidative stress in yeast *Saccharomyces cerevisiae* by promoting ROS production. BPA-induced ROS are formed not only in the cytoplasm, but mainly in the mitochondria. Moreover, we show that the nuclear genome integrity and protein carbonylation level are virtually not affected by BPA treatment. We provide strong evidence that BPA likely affects the proper function of the mitochondria in yeast cells, while the protein oxidation level and nuclear DNA remain largely unaffected.

## 5. Discussion

Despite the large quantity of information about the effects of BPA on living organisms, especially about its estrogenic activity, relatively little is still known about its non-estrogenic activities. In our previous study [44], a DNA topology assay was used to demonstrate that BPA had no damaging effects on naked plasmid DNA. Therefore, we assumed that BPA may not have a mutagenic effect. However, we hypothesized that upon entry into a cell, BPA may pose a potential risk due to cell metabolism, for example, due to the induction of oxidative stress, adversely affecting the cell nucleus and/or mitochondria. Moreover, our previous findings [29] pointed to excessive ROS production after 500 µM BPA treatment in *Schizosaccharomyces pombe* cells that did not contain ERs. Oxidative stress in this type of cell leads to cell cycle arrest. As oxidative stress can also cause genotoxic effects, we focused on determining the origin of ROS production within the cellular context, together with an investigation of the nuclear genome integrity of *S. cerevisiae* cells. Therefore, we assessed whether DSBs was induced after BPA treatment and whether mitochondria were involved in cellular response to BPA exposure.

To evaluate the toxicity of BPA, we monitored cell growth rate and cell survival after treatment with this compound. The results showed that BPA manifested toxicity and slowed down the growth rate only at concentrations higher than or equal to 100 µM.

Since ROS can attack important macromolecules, including DNA, and thus cause oxidative damage leading to DNA strand breakage [42], we examined DSB induction after BPA treatment in yeast cells. DSBs may arise in DNA directly or indirectly as a consequence of cellular metabolism, for example, ROS can damage DNA bases causing lesions that can block the progression of DNA replication, resulting in DSB formation. In this study, a short-time exposure to high(er) BPA concentrations did not cause DSB formation in the yeast nuclear genome. The observed subtle DSB breakage at high(er) BPA concentrations may suggest that part of the toxic effects mediated by this compound involve DSBs generated in chromosomal DNA. Furthermore, the observed slightly increased DSB breakage after treatment with the Fpg enzyme, which recognizes oxidized purines, may indicate that BPA can also induce oxidative base damage to chromosomal DNA. To the best of our knowledge, this is the first evidence that BPA induces oxidative base damage to DNA, which is the substrate for Fpg, in yeast cells. Accordingly, a long-term exposure to BPA induces DNA damage in ovarian cells in rats, as evidenced by increased γH2AX and ATM levels [45]. ATM is a protein kinase that phosphorylates histone H2AX producing γH2AX within a few seconds after DSB induction, thereby, recruiting DSB repair proteins to the break site [46]. In addition, according to Liu et al. [47], adult male exposure to BPA induced persistent DSBs in pachytene spermatocytes and disrupted meiotic progression in rats. In that case, however, the mechanism of action was attributed to the estrogen-like activity of BPA [47], not to oxidative stress. In the present study, the use of yeast cells as a model organism dissected the effects of BPA, allowing us to demonstrate that BPA can induce ROS by a mechanism that is not dependent on estrogen receptors.

Herein, ROS levels in living yeast cells after BPA treatment were measured by flow cytometry and fluorescence microscopy after staining with DCFDA. We report that 1000 µM BPA effectively induces ROS in *S. cerevisiae* cells, resulting in oxidative stress, an observation that is in line with our previous findings obtained in fission yeast [29]. In addition, we show that this treatment also increases mitochondrial O_2_^•−^ levels. Dragone et al. [48] studied the mitochondrial interference of BPA and bisphenol B (BPB) (5–100 µg/mL) in the yeast *S. cerevisiae* following long-term (24 h) exposure and revealed a similar hyperstimulation effect at 5 µg/mL concentration for BPA and BPB, respectively. However, about two-fold higher cellular respiration inhibition potency was observed for BPB as compared with BPA after exposure to 15, 30, and 100 µg/mL. Moreover, a time-dependent mechanism of mitochondrial interference was highlighted. However, our results show that BPA is able to induce oxidative stress in the mitochondria in a much shorter time, even after 3 h exposure. ROS overproduction may lead to irreversible damage of membrane lipids and proteins, resulting in detrimental effects on the mitochondrial structure and function, including aerobic respiration [46]. In addition, BPA intake has been shown to significantly reduce the expression of the mitochondria-encoded genes and mitochondrial DNA copy numbers in rat colon and liver [49]. The putative mechanism of BPA action in mitochondria may be a decrease in the activity of enzyme complexes of the electron transport chain. Enzymes of complex I and complex III are involved in mitochondrial ROS generation by forward and reverse electron transports [50]. The decreased activity of complexes I and III caused by BPA treatment may induce excessive ROS production, and therefore may lead to oxidative damage to mitochondria, which may affect oxidative phosphorylation with further alterations in mitochondrial respiration [31].

Moreover, cells lacking mtDNA (ρ^0^) displayed unchanged ROS levels after exposure to the whole concentration range of BPA. In contrast, cells with mtDNA (ρ^+^) showed a dose-dependent increase in ROS levels, suggesting mitochondrial involvement in ROS production in *S. cerevisiae* after BPA treatment.

Thereafter, we evaluated MMP using MitoTracker Red probe and fluorescence microscopy. MitoTracker Red passively crosses the plasma membrane and subsequently accumulates in the mitochondria of metabolically active cells, in particular, in their negatively charged matrix [51]. The extent of dye absorption into the mitochondria depends on its membrane potential, with a decrease in membrane potential resulting in a decrease in the intensity of the fluorescent signal. Thus, the use of MitoTracker Red in conjunction with fluorescence microscopy can serve as a reliable tool for detecting changes in the membrane potential of mitochondria [51]. These changes may also serve as a marker of apoptosis [52], which is characterized by depolarization and changes in membrane permeability and the associated release of apoptotic factors [53]. Our results show the ability of BPA to reduce MMP. Acute exposure to 100 µM BPA leads to a decrease of more than one-third as compared with the control. Moreover, we observed a toxic effect of 1000 µM BPA, which was manifested by morphological changes of mitochondria. Similarly, Kaur et al. [54] observed a significant decrease in the MMP of lymphoblastoid cells isolated from children diagnosed with autism. According to Huang et al. [55], a high concentration of BPA significantly decreased the MMP in KGN cells, accompanied by increased Ca^2+^ levels.

To further evaluate the oxidative potency of BPA in an intracellular environment, we monitored the protein carbonyl content after BPA exposure. We demonstrated virtually no ability of BPA to alter the protein carbonyl content in *S. cerevisiae* cells. The levels of protein carbonylation were also investigated on mice hepatic microsomes, mitochondria, and cytosol, 72 h after BPA administration per os [56]. Shmarakov et al. [56] found that administration of 50 mg/kg BPA caused an increase in the protein carbonyl content in microsomal and cytosolic fractions derived from mice liver. Hence, it seems that the BPA effect on this oxidation parameter may be dependent on the cell type or length of exposure. Alternatively, and very speculatively, it may be ER-dependent.

## Supplementary Materials

The following are available online at https://www.mdpi.com/article/10.3390/jof7070543/s1, Figure S1: The protein carbonylation after acute BPA and SeL treatment in *S. cerevisiae* cells, Figure S2: Total protein content, Figure S3: The level of carbonylation in BPA- or SeL-treated samples.
