# Supplementary figures and images for: Acute Exposure to Bisphenol A Causes Oxidative Stress Induction with Mitochondrial Origin in Saccharomyces cerevisiae Cells
Source: J Fungi (Basel). 2021 Jul 7;7(7):543. doi: 10.3390/jof7070543 (PMC8303452; doi:10.3390/jof7070543)

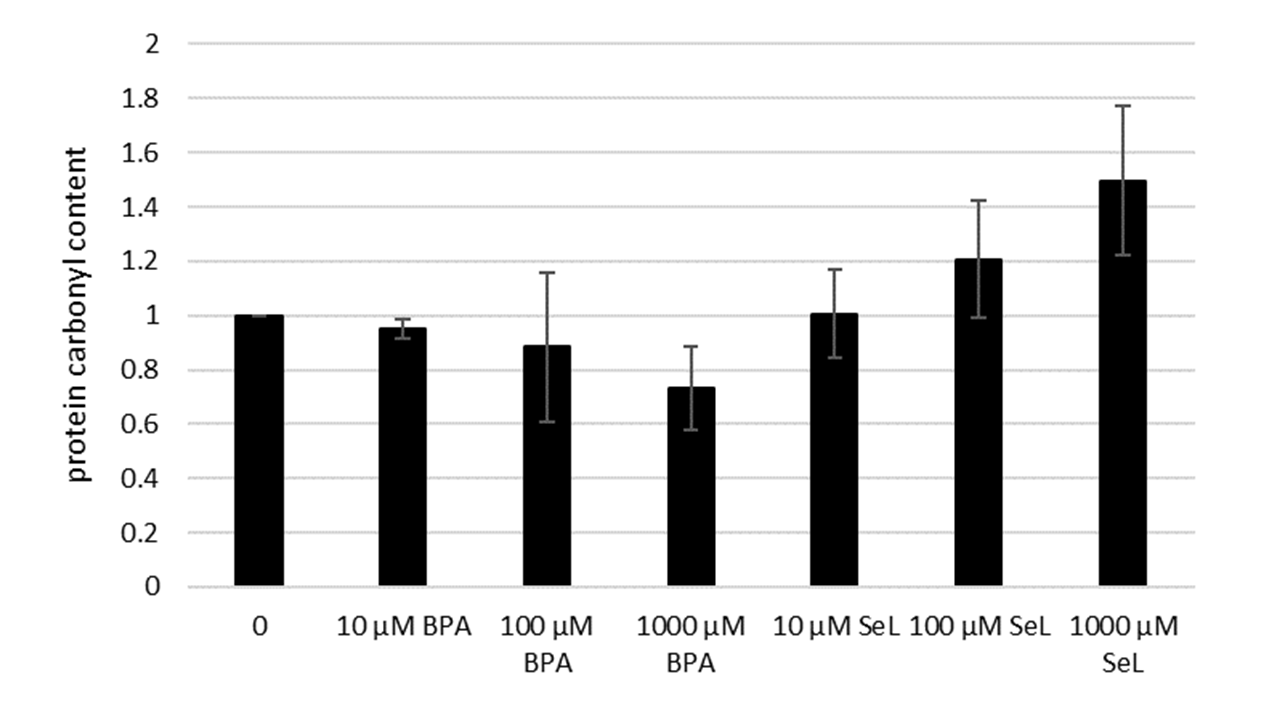

Supplement: Supplementary file 1 [file jof-07-00543-s001.zip › jof-1296879-supplementary/jof-1296879-supple/Figure S1 The protein carbonylation after acute BPA and SeL treatment in S. cerevisiae cells.tif]

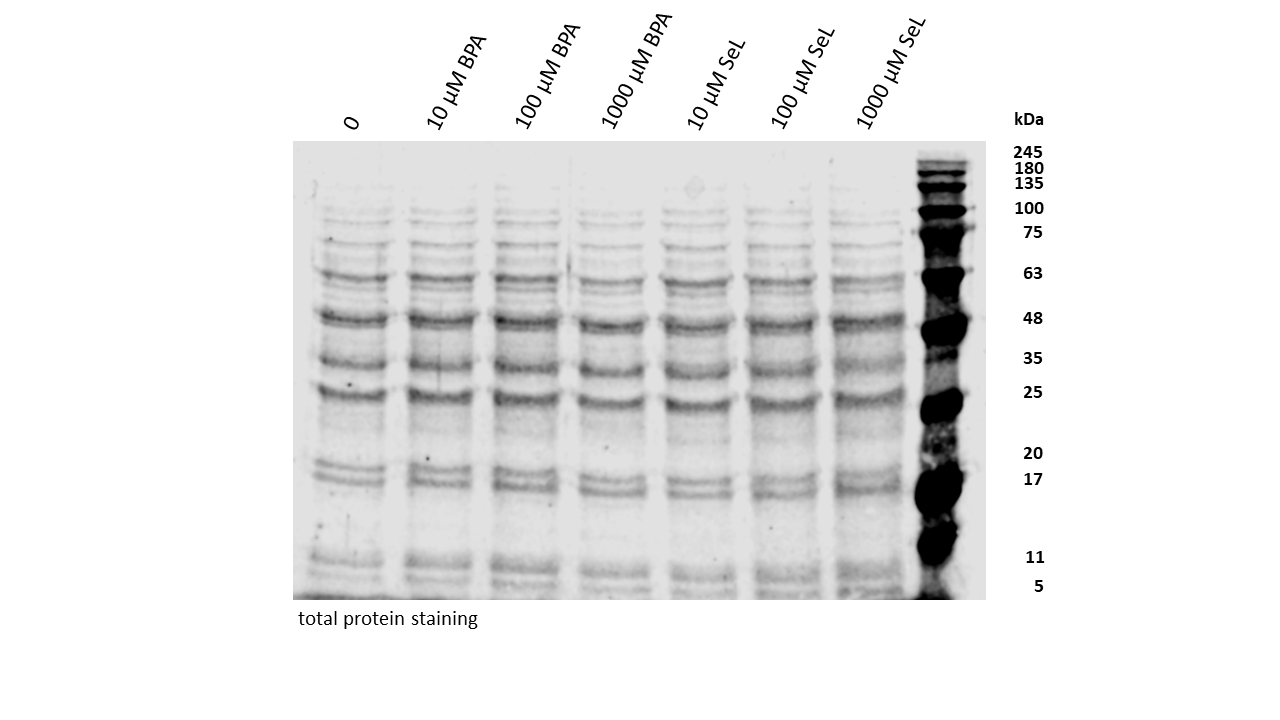

Supplement: Supplementary file 1 [file jof-07-00543-s001.zip › jof-1296879-supplementary/jof-1296879-supple/Figure S2 Total protein content.tif]

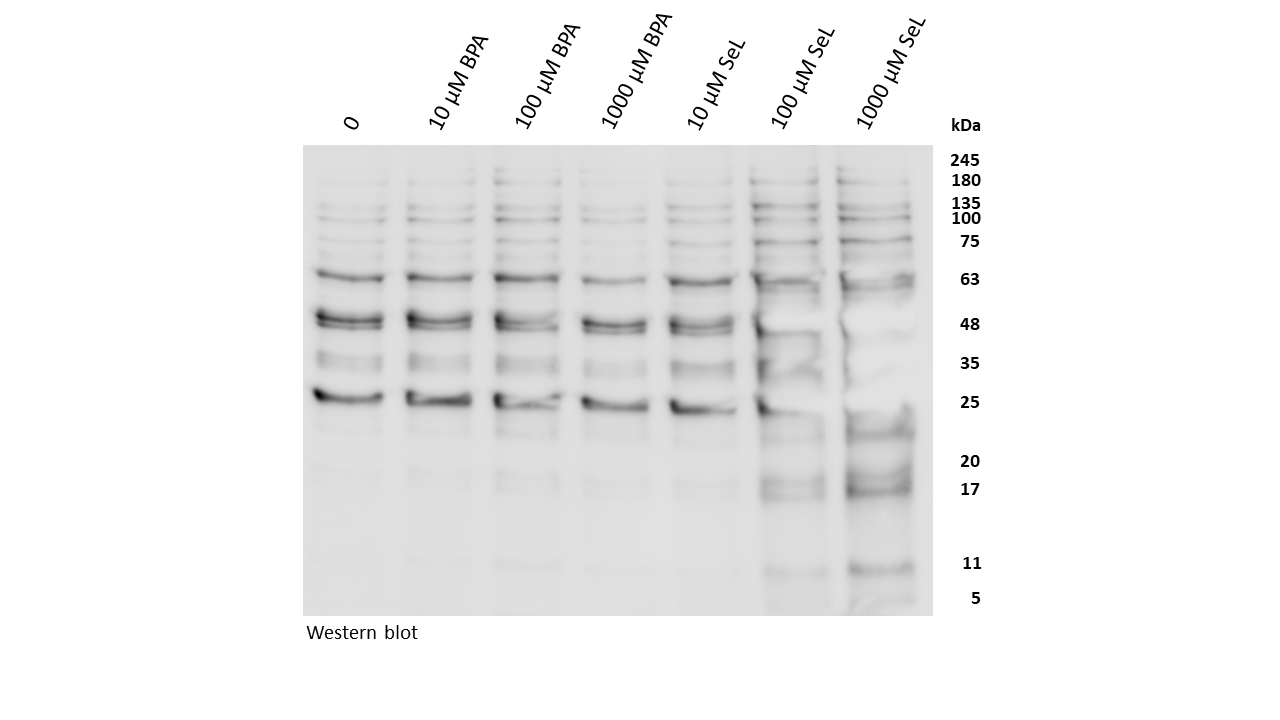

Supplement: Supplementary file 1 [file jof-07-00543-s001.zip › jof-1296879-supplementary/jof-1296879-supple/Figure S3 The level of carbonylation in BPA- or SeL-treated samples.tif]
